# Editorial: Vascular Dysfunction Beyond Pathological Pregnancies. An International Effort Addressed to Fill the Gaps in Latin America

**DOI:** 10.3389/fphys.2019.00950

**Published:** 2019-07-24

**Authors:** Fernanda Regina Giachini, Carlos Galaviz-Hernandez, Alicia E. Damiano, Carlos Escudero, RIVA-TREM

**Affiliations:** ^1^Laboratory of Vascular Biology, Institute of Biological Sciences and Health, Federal University of Mato Grosso, Barra do Garças, Brazil; ^2^Academia de Genómica, Instituto Politécnico Nacional-CIIDIR Durango, Durango, Mexico; ^3^Laboratorio de Biología de la Reproducción, IFIBIO Houssay-UBA- CONICET, Buenos Aires, Argentina; ^4^Cátedra de Biología Celular y Molecular, Departamento de Ciencias Biológicas, Facultad de Farmacia y Bioquímica, Universidad de Buenos Aires, Buenos Aires, Argentina; ^5^Group of Investigation in Tumor Angiogenesis, Group of Research and Innovation in Vascular Health (GRIVAS Health), Vascular Physiology Laboratory, Department of Basic Sciences, Universidad del Bío-Bío, Chillán, Chile

**Keywords:** vascular, dysfuction, Latin America, pregnancy, pathological pregnancy

Pregnancy is a physiologically stressful condition that generates a series of functional adaptations in the cardiovascular system. Recent evidence suggests that vascular changes associated with pregnancy complications may impair the function of the maternal and offspring vascular systems after delivery, being possibly extended until adult life.

In Latin American countries, like other low (LIC) and middle-income countries (MIC) worldwide, the rate of morbi-mortality due to both pregnancy complications and cardiovascular diseases have a higher incidence than in high-income countries. Paradoxically, research in this field is limited in Latin America (Giachini et al., 2017). Then, in addition to the scientific and public health implications of the maternal morbi-mortality in LIC and MIC, we also aimed to overcome geographic limitations. Therefore, our Research Topic titled “Vascular Dysfunction Beyond Pathological Pregnancies. An International Effort Addressed to Fill the Gaps in Latin America” intends to positively contribute in the scientific field, but also to visualize the challenging need for more investigation in our countries.

A highly diverse human population is observed within Latin America, and then many, risk factors for pregnancy complications are present in Latin American women. For example, the evaluation of genetic variants is critical to identify candidate genes that may contribute to the pathophysiology of pre-eclampsia in any specific population. Michita et al. have provided an integrative view of the genes evaluated by Latin American research groups, displaying a specific role on different aspects related to pre-eclampsia. They also discussed important topics related to pregnancy vascular disorders, which may be related to pre-eclampsia development, including epigenetics, transplantation biology, and non-coding RNAs.

Another interesting aspect on the pathophysiology of pre-eclampsia is that not only the mother, but also the father may be involved in the early onset of the disease, as revised by Galaviz-Hernandez et al. Indeed, the existence of a paternal antigen in the placenta has already been proposed in the specialized literature. For instance, evidences of paternal contribution include nulliparity, number of partners, among others, are remarked in the Galaviz-Hernandez et al. manuscript. Interestingly, not only maternal but also paternal obesity is a risk factor for pre-eclampsia.

Maternal nutritional condition is a key component in normal pregnancy development; and malnutrition by excess constitutes another increasing risk factor to pregnancy morbidity including pre-eclampsia. Despite that, as Lopez-Jaramillo et al. remark, not only obesity increases the risk of pre-eclampsia, but also some nutritional deficiencies of essential elements, predispose the mother to suffer pregnancy complications. On this regard, Lopez-Jaramillo et al. have discussed the alterations in the L-arginine/nitric oxide pathway that are commonly observed during obesity and may represent a key element in pre-eclampsia.

Another aspect related to nutritional deficiencies as well as obesity in pregnancies is the oxi-redox balance. In this regard, Alcala et al. review current evidences related with the negative impact of obesity (a well-characterized low-state chronic inflammation and high oxidative stress condition) to generate an unhealthy environment that predispose to development of adverse outcomes during gestation. Nevertheless, they also discuss the controversial results on antioxidant supplementation as a therapeutic tool during obese pregnancies.

Another risk factor to pathological pregnancies is the antiphospholipid syndrome (APS), a well-known condition linked with endothelial dysfunction. Velásquez et al. have compared the current understanding about the mechanisms of endothelial dysfunction induced by patient-derived anti-phospholipids auto-antibodies (aPL) under the two main clinical manifestations of APS: thrombosis and gestational complications, either alone or in combination. Analyzing current evidences in the field, Velásquez et al. challenge the current knowledge proposing that the mechanism of aPL-induced endothelial dysfunction depends on clinical manifestation of APS.

Placentation is clearly a key process for normal pregnancy development and many groups in Latin America have studied the placenta function. Indeed, the Latin American Society for Materno Fetal Interaction and Placenta (SLIMP) agglutin groups of researchers in this field. Teran et al. analyze the role of coenzyme Q10 (CoQ10) in placentation during pre-eclampsia. In their manuscript, extend the well-described role of CoQ10 as antioxidant and part of the mitochondria respiration chain into an effective intervention to reduce pre-eclampsia occurrence in Ecuadorian pregnant women.

We also highlight participation of other components such as aquaporins (AQPs) in the placentation process. On this regard, Szpilbarg et al. explain us how AQPs, a family of proteins that are known to work as water channel proteins, may display an additional role in the cellular homeostasis. This family of proteins also participates in cell signaling process including migration and apoptosis, which in turn are remarked component of normal placentation. Szpilbarg's manuscript details how a defective expression and activity of AQPs may result in the characteristic impaired placentation and systemic endothelial dysfunction underlying pre-eclampsia.

Continuing with analysis of placentation, Abán et al., provide current evidences about the interactions between endocannabinoids (ECS) and nitrergic signaling pathways during normal and pathological placentation process, observed both in intrauterine growth restriction and pre-eclampsia. In particular, ECS (a group of lipid-signaling molecules that include amides, esters and ethers of long-chain polyunsaturated fatty acids) can activate cannabinoid receptors, such as CB1 and CB2, leading to generation of nitric oxide (NO). Despite current knowledge about the relationship between ECS and NO synthesis, the underling molecular mechanisms, as well as its implications for abnormal placentation are still unclear.

In addition, Lima et al. present their work related with disturbances in the polysaccharide metabolism that may result in intracellular saccharide deposition, modulating cellular function*. O-*GlcNAcylation is a reversible post-translational modification that has been implicated as a modulator of protein function, both in physiological and pathological conditions including those in placental tissue. The interplay between *O-*GlcNAcylated placental proteins and the possible implications of this post-translational modification through placental development and pregnancy were also discussed.

Alterations in pregnancy due to pre-eclampsia, intrauterine growth restriction, or any other placental alterations are linked to systemic endothelial dysfunction, which then has profound implications in future cardiovascular health in both mother and her children. We started this analysis with the Galvis-Ramírez et al. manuscript, who reviewed the role of the structural domains of heparin sulfate (HS) in the process of selective permeability through the glomerular filtration barrier (GFB) and how these domains may be implicated in the glomerular inflammation processes observed in pre-eclamptic pregnancies.

Another key biological barrier forming by endothelial cells is the blood brain barrier (BBB), a tightly sealed monolayer of brain microvascular endothelial cells characterized by absence of fenestrations, low number of pinocytic vesicles and junctional complex formed by tight junctions and adherent junctions. In particular, it is known that the majority of maternal deaths resulting from pre-eclampsia are attributed to the coexistence of neurological complications. Torres-Vergara et al. help us to better understand this process by reviewing preclinical studies related to how the BBB is impaired in pre-eclampsia predisposing to cerebral edema and therefore brain complications in the mother not only during her pregnancy but even years after that.

Following brain complications and extending to offspring, Lara et al. propose a challenging hypothesis about how pre-eclampsia might impair brain angiogenesis in the offspring. In particular, they speculate how angiogenesis, as a key event for favoring the correct neurodevelopment and function could be disrupted in children born to pre-eclampsia. Proposed mechanism for impaired brain angiogenesis should consider imbalance of pro-angiogenic factors, including the vascular endothelial growth factor (VEGF) and placental growth factor (PlGF), with anti-angiogenic factors such as soluble VEGF receptor type 1 (or Flt-1).

In conclusion, although there are obvious deficiencies in terms of economies and scientific infrastructure between countries, Latin American researchers have been able to generate milestone knowledge and contribute in the better understanding of vascular alterations present in pregnancy complications, indeed, it is the spirit of the Iberoamerican consortium called RIVA-TREM (Red Iberoamericana de alteraciones Vasculares Asociadas a TRastornos del EMbarazo).

We challenge ourselves to continuing our effort to visualize productivity and more important potential geographic and cultural particularities that are partially considered in the specialized literature. Then, we would like to encourage vascular biology researchers in Latin America that have continuing contributing to both better understand vascular dysfunction associated to pregnancy diseases and show the gaps in the literature, to overcome this hidden effect of our scientific production (Alperin, 2014; Van Noorden, 2014). This effort also will homogenize clinical concepts and knowledge that may strength the scientific effort in Latin America focused in reducing maternal and fetal morbi-mortality in our countries.

## Author Contributions

All authors listed have made a substantial, direct and intellectual contribution to the work, and approved it for publication.

### Conflict of Interest Statement

The authors declare that the research was conducted in the absence of any commercial or financial relationships that could be construed as a potential conflict of interest.
